# Supplementary material for: Attenuating Mutations in Usutu Virus: Towards Understanding Orthoflavivirus Virulence Determinants and Live Attenuated Vaccine Design
Source: Vaccines (Basel). 2025 May 3;13(5):495. doi: 10.3390/vaccines13050495 (PMC12115599; doi:10.3390/vaccines13050495)
Supplement: Supplementary file 1 [file vaccines-13-00495-s001.zip › vaccines-3557754-supplementary.pdf]

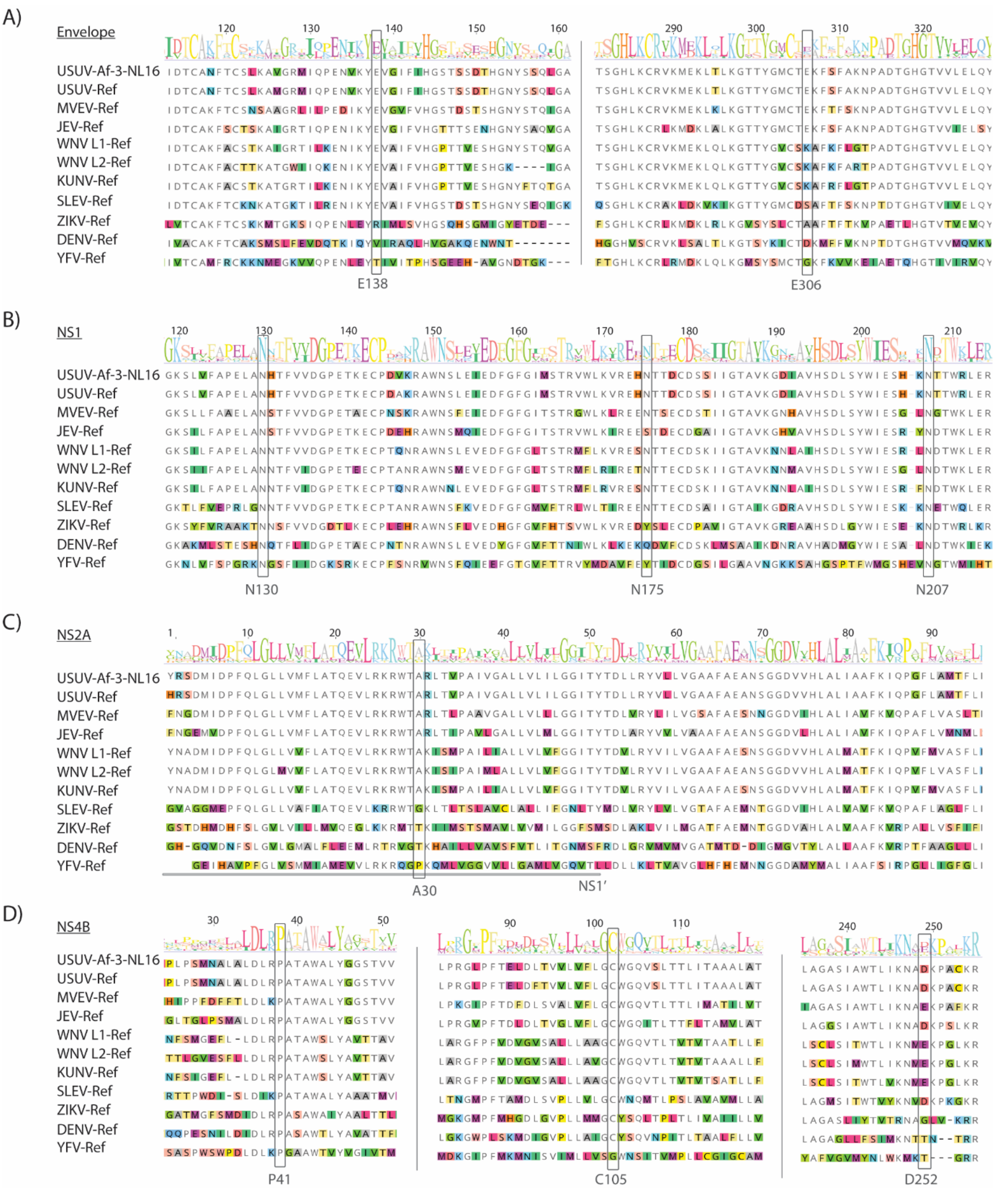


Figure S1. Conservation of the regions that were mutated in USUV in related orthoflaviviruses.

Amino acid sequence alignments between USUV-Af3 (NL 2016 strain) and selected related Orthoflaviviruses marking the sites of inserted amino acids changes for A) Envelope, B) NS1, C) NS2A (overlap with NS1' protein is also depicted) and D) NS4B. Consensus is depicted above in a sequence logo numbered by amino acid location, and coloured by individual amino acid. In each sequence the disagreements to the consensus are highlighted. Alignments and consensus were made in Geneious

version 10.2 created by Biomatters. Available from https://www.geneious.com. Genbank reference numbers for sequences used in alignment can be found in Supplemental Table 4.

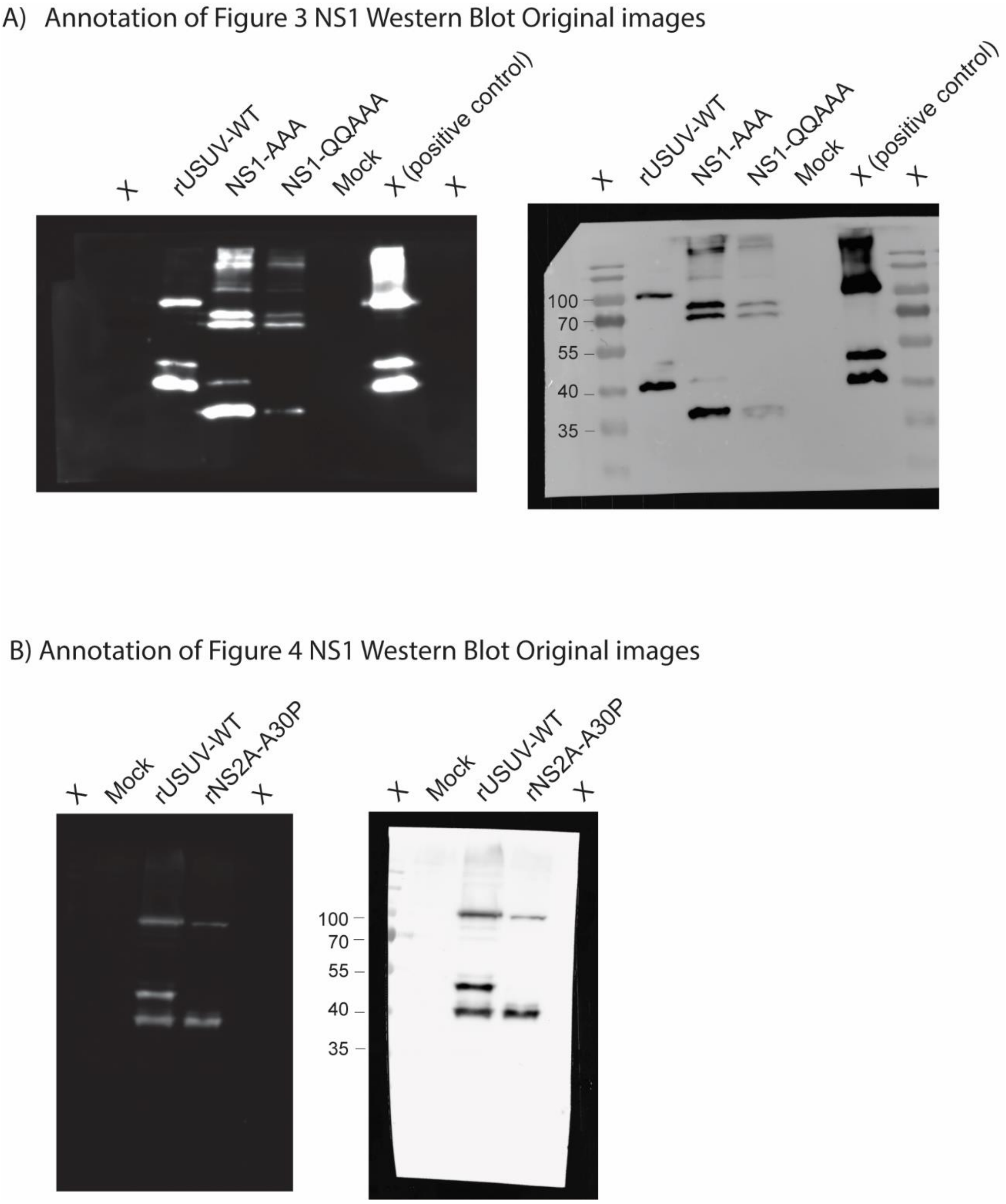


Figure S2. Western blot raw data with annotations.

Uncropped annotated images of western blots used in A) Figure 3 and B) Figure 4, with and without the ladder marker. X denotes lanes not shown in the figures.

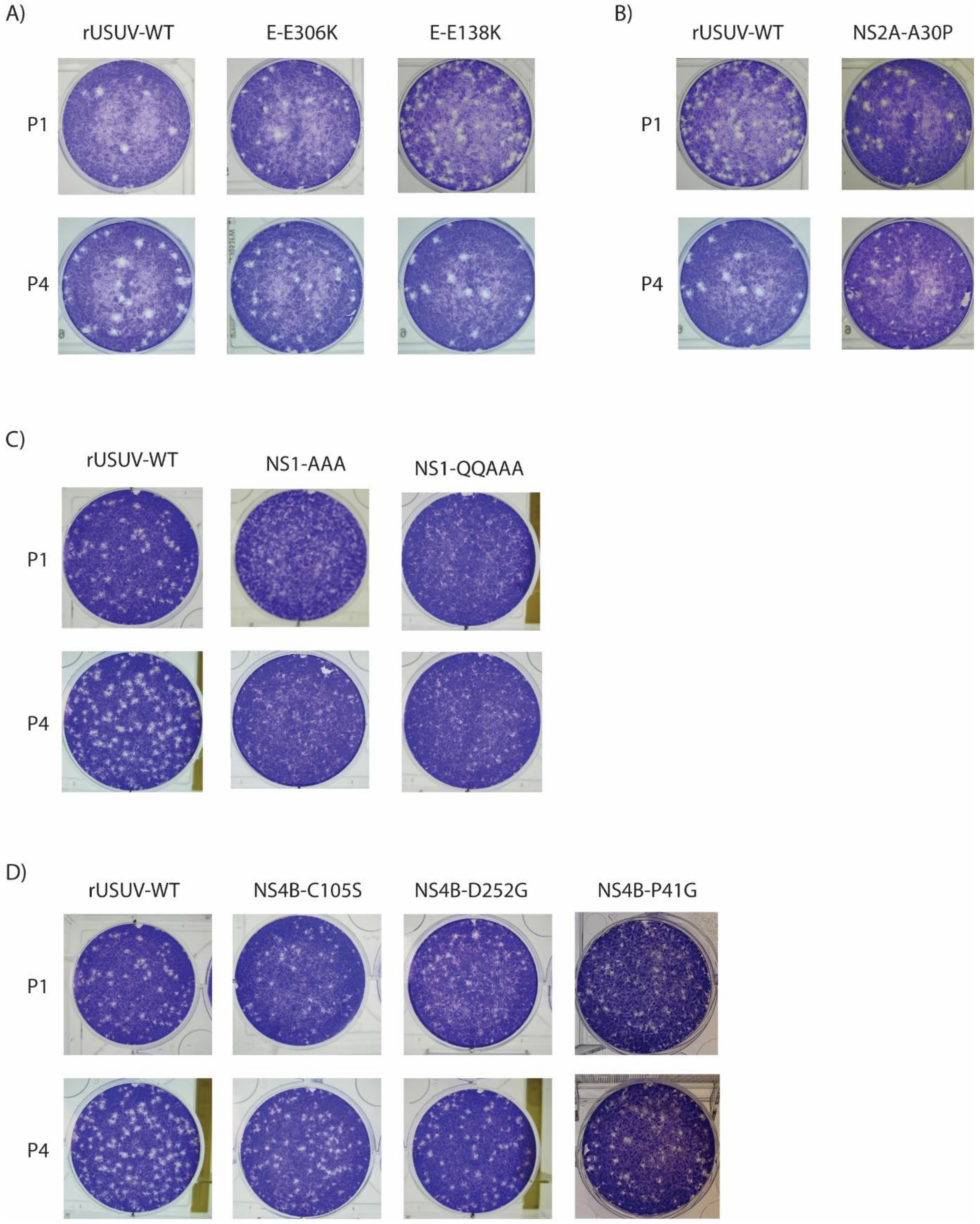


Figure S3. Plaque assay phenotypes of mutant USUV viruses at passage1 and passage4.

Virus stocks of mutant viruses were harvested after launch and grown on Vero CCL-81 cells for one passage (P1), or passaged a further 4 times (P4)to check stability. Plaque assays were performed on BHK21-J cells and. A representative image of the stained well is shown for each virus and a rUSUV-WT control, at both passages, for A) Envelope mutants, B) NS2A mutant, C) NS1 mutants and D) NS4B mutants.

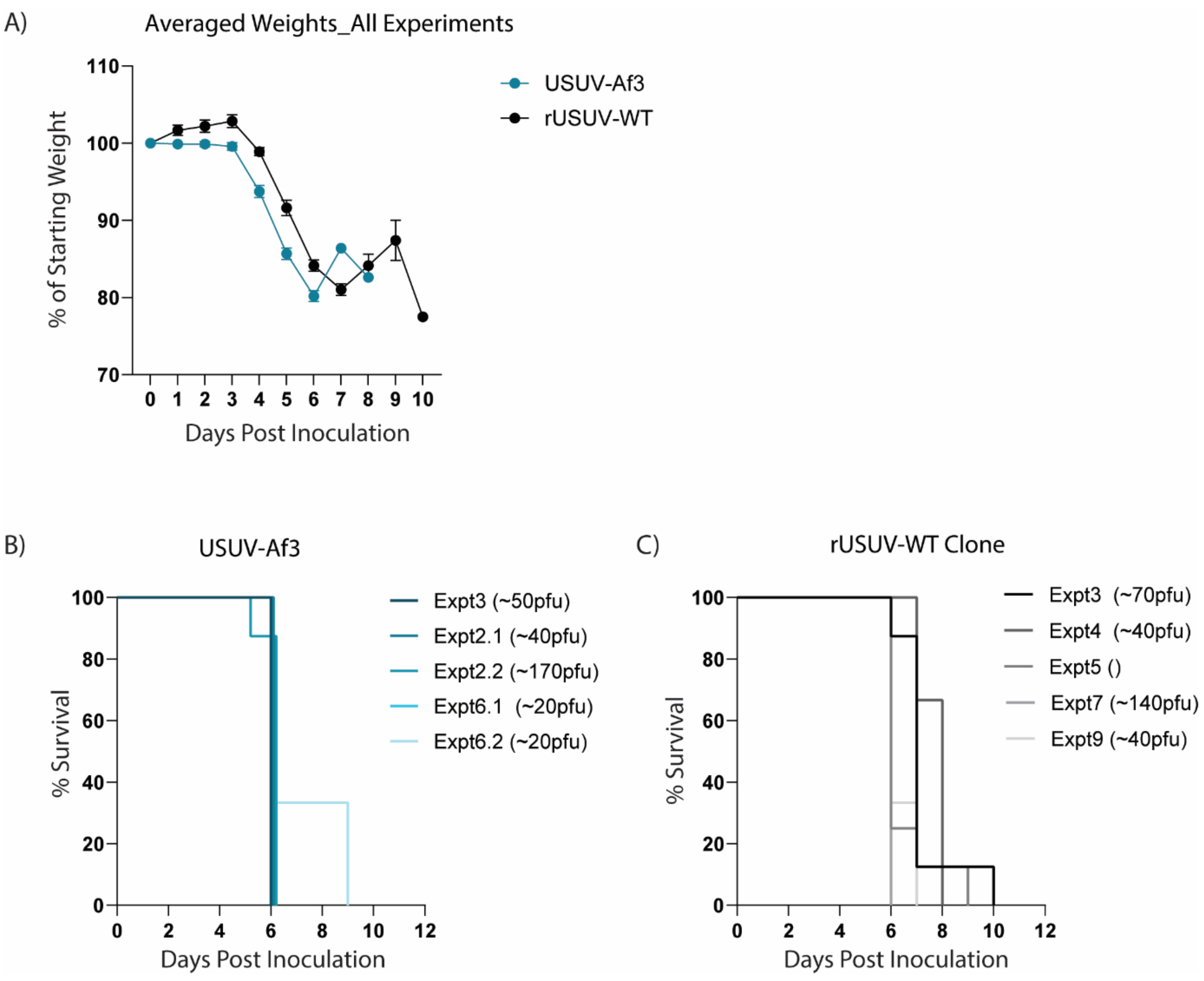


Figure S4. Comparison of recombinant clone-derived USUV to its corresponding natural isolate in replicate mouse experiments.

Ifnar$^{-/-}$ mice were inoculated SC with 20 or 100 pfu/mouse of USUV-Af3 virus or clone-derived rUSUV-WT virus. Animals were weighed daily and euthanised when they reached humane endpoint. A) Daily weight loss measured as a percentage of initial weight for each of the experimental groups showing mean ± SD. B) and C) Survival rates for USUV-Af3 natural isolate virus or clone-derived rUSUV-WT virus respectively. Statistical analysis was performed using the log-rank (Mantel-Cox) test. The experiment number and the USUV pfu/mouse titres from back titrations of virus inoculum are shown in the figure legend.

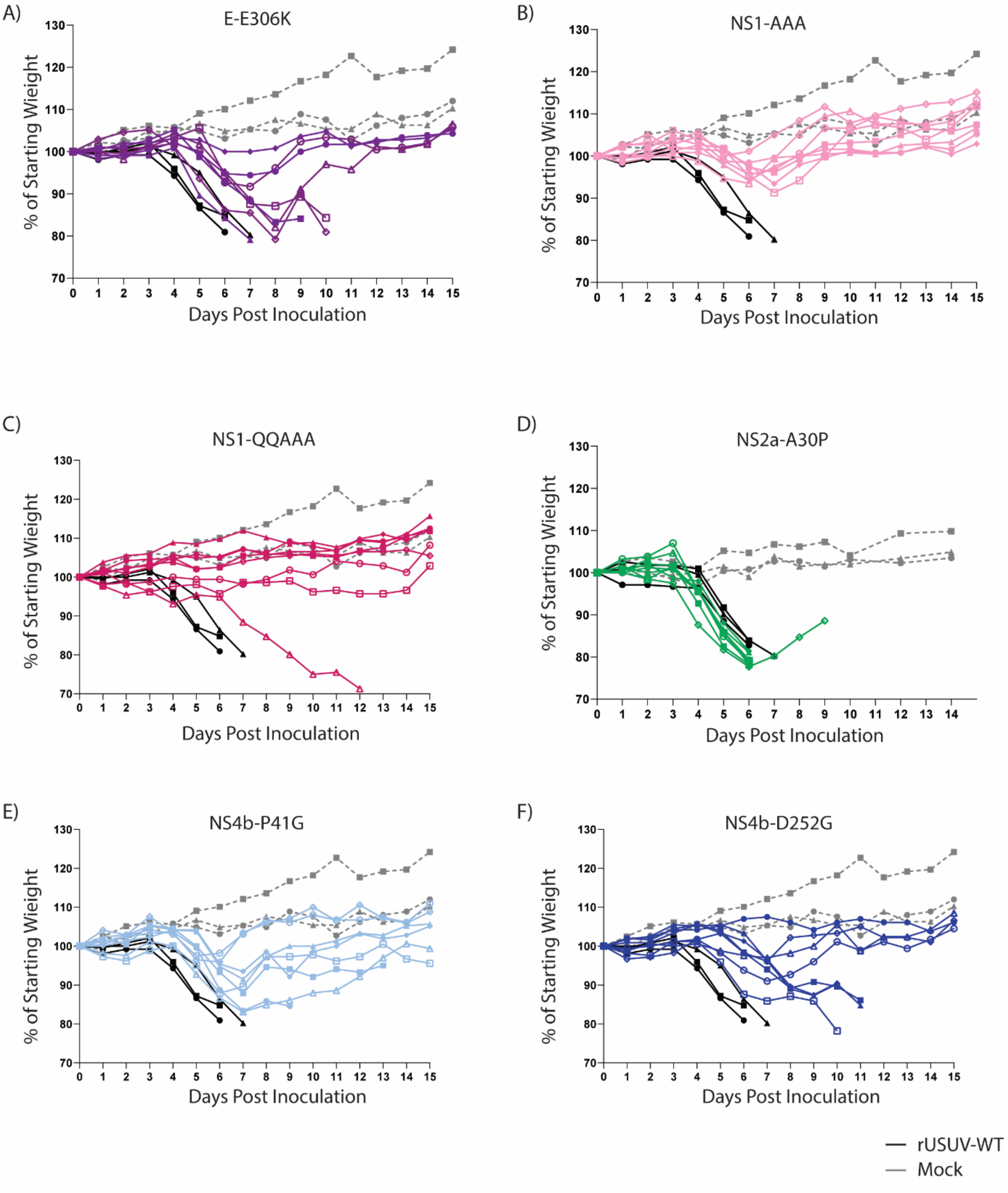


Figure S5. Individual weights for mutant virus inoculated Ifnar$^{-/-}$ mice.

Ifnar$^{-/-}$ mice were inoculated subcutaneously (SC) with DMEM alone, with 1.4 x10$^{2}$ TCID50/mouse of clone-derived rUSUV-WT virus or 1.4 x10$^{2}$ TCID50/mouse of each mutant virus (or 100 Pfu/mouse for the NS4B-P41G mutant). Animals were weighed daily and euthanised when they reached humane endpoint or at day 16, the end of experiment. Daily weight loss for each mouse measured as a percentage of initial weight for each of the experimental groups E-E306K (A), NS1-AAA (B), NS1-QQAAA (C), NS2A-A30P (D), NS4B-P41G (E) and NS4B-D252G (F) is compared to weight loss of

individuals from the mock (grey, dotted line) and rUSUV-WT (black) groups. For each group of mutant infected mice, males (mouse 1-4 per group) are depicted as filled shapes (circle, square, upright tringle and downfacing triangle respectively), and females (mouse 5-8 per group) are depicted as outline shapes (circle, square, upright tringle and downfacing triangle respectively).

## Table S1. Primers used in this study

A) Primers used in mutant Site Directed Mutagenesis

| Gene | Amino Acid Change(s) – Gene # | Nucleotide Change(s) – Genome # | Sequences (Fwd/Rev) |
|---|---|---|---|
| E | E138K | G1387A, A1389G | CCATGTATGAAGATTCCCACCTTATACTTAACATTTTCCGGTTG<br>CAACCGGAAAATGTTAAGTATAAGGTGGGAATCTTCATACATGG |
| | E306K | G1891A, A1893G | GATTTTTTGCAAAAGAAAACTTCTTCGTACACATGCCGTAGGTGG<br>CCACCTACGGCATGTGTACGAAGAAGTTTTCTTTTGCAAAAAATC |
| NS1 | N130A | A2863G, A2864C | CGCACCAGAACTGGCCGCCCACACTTTTGTGGTTG<br>CAACCACAAAAGTGTGGGCGGCCAGTTCTGGTGCG |
| | N175A | A2998G, A2999C | GAAAGTCAGAGAGCACGCCACTACTGACTGCGACAGCTC<br>GAGCTGTCGCAGTCAGTAGTGGCGTGCTCTCTGACTTTC |
| | N207A | A3034G, A3095C | GATTGAAAGCCACAAGGCCACGACATGGAGGCTCGAG<br>CTCGAGCCTCCATGTCGTGGCCTTGTGGCTTTCAATC |
| | N130Q, H131Q T132A | A2863C, C2865G, C2868G, A2869G, T2871G | CACCAGAACTGGCCCAGCAGGCGTTTGTGGTTGACGG<br>CAACCACAAACGCCTGCTGGGCCAGTTCTGGTGCG |
| NS2A | A30P | G3619C | GAGGAAGAGGTGGACGCCCAGATTGACTGTTCCGG<br>CCGGAACAGTCAATCTGGGCGTCCACCTCTTCCTC |
| NS4B | P41G | C7030G,C7031G | CATTGGATTTGCGAGGAGCAACAGCGTGGGCCTTATAC<br>GTATAAGGCCCACGCTGTTGCTCCTCGCAAATCCAATG |
| | C105S | G7223C | GTCTTGGTCTTTTTGGGATCCTGGGGCCAAGTGTCGTTAAC<br>GTTAACGACACTTGGCCCCAGGATCCCAAAAAGACCAAGAC |
| | D252G | A7664G | CTGATAAAGAATGCTGGCAAACCGGCCTGCAAACGAG<br>CTCGTTTGCAGGCCGGTTTGCCAGCATTCTTTATCAG |

B) Primers used in USUV qRT-PCR protocol

| Primer | Sequence |
|---|---|
| USUV _Fwd | TCAGAAAAGACGTGCCAGAG |
| USUV_Rev | AAAGTCCTTCCGTCCTTCATG |
| USUV_Probe_FAM | CCTGAAAGTGGTTTGAGCAGAAAGGC |
| EAV_Fwd | CATCTCTTGCTTTGCTCCTTAG |

| | |
|---|---|
| EAV_Rev | GCTTTGCCATTGGGTTGATACC |
| EAV_Probe_TQ-CY5 | CGCTGTCAGAACAACATTATTGCCCAC |

C) Primers used cDNA synthesis, PCR amplification and sanger sequencing for mutant specific regions

| **Primer** | **Sequence** |
|---|---|
| E_E306K_Fwd | AGTGTAAAGCTGACATCTGG |
| E_E306K_Rev | CAGCACTTTCGCGTTAGCTT |
| NS1_AAA_Fwd | TCAGCACCGCAGAGATTAGC |
| NS1_AAA_Rev | CCAGATCACTTTCAACAACG |
| NS2A_A30P_Fwd | GTGCCTACCGGAGTGACATG |
| NS2A_A30P_Rev | GGCCAGCAGGATGTTCTCTT |
| NS4B_P41G_Fwd | TCAGCTGTGGGATTCCTTG |
| NS4B_P41G_Rev | GCTGCCGCAGTAATCAGAG |
| NS4B_D252G_Fwd | ACAACGGAGCCATTGCAGTA |
| NS4B_D252G_Rev | AAATCCTCCTTGCTGAGCCC |

## Table S2. Titration of viral inoculum used in animal experiments

A) Back titrations for in vivo experiment one (rAf-3-WT)

| Group | PFU/ml: Expected | PFU/ml: Measured | PFU/Mouse |
|---|---|---|---|
| USUV-Af3 virus | 1000 | 4.8E+02 | 48 |
| rUSUV-WT | 1000 | 6.6E+02 | 65 |

B) Back titrations for in vivo experiment two (Mutants.A)

| Group | PFU/ml: Expected | PFU/ml: Measured | PFU/Mouse |
|---|---|---|---|
| rUSUV-WT | 1000 | 375 | 37.5 |
| NS2A-A30P | 1000 | 315 | 31.5 |

C) Back titrations for in vivo experiment three (Mutants.B)

| Group | Titre/ml: Expected | Titre/ml: Measured | Titre/Mouse |
|---|---|---|---|
| rUSUV-WT | 1.40E+04 TCID50 | 1.0E+05 TCID50 | 1,0E+04 TCID50 |
| E-E306K | 1.40E+04 TCID50 | 3.2E+05 TCID50 | 3,2E+04 TCID50 |
| NS1-AAA | 1.40E+04 TCID50 | 2.2E+04 TCID50 | 2,2E+03 TCID50 |
| NS1-QQAAA | 1.40E+04 TCID50 | 1.0E+05 TCID50 | 1,0E+04 TCID50 |
| NS4B-P41G | 1.00E+02 PFU | 9.9E+02 PFU | 9,9E+01 PFU |
| NS4B-D252G | 1.40E+04 TCID50 | 2.2E+04 TCID50 | 2,2E+03 TCID50 |

Table S3. Complete sequencing results of Bacmid (prelaunch) and passage 4 virus stock compared to rUSUV-WT.

Key: Green highlight – Rationally Designed Site Mutation. Orange Highlight – Reversion of inserted mutation, Yellow highlight – Partial reversion of inserted mutation.

*Mutations are present in sequencing results across different mutants.

E-E138K

| Protein | Pre-Launch: Nucleotide Change | Amino Acid Change | Passage 4: Nucleotide Change | Amino Acid Change |
|---|---|---|---|---|
| M | | | T936G (7.3%)* | na |
| M | | | C946G (5.4%)* | M - L66V |
| M | | | C952A (5.5%)* | M - L68I |
| E | G1387A | E - E138K | G1387A (98.6) | E - E138K |
| E | A1389G | | A1389G (99.6) | |
| NS1 | | | C3349 +A (10.4%) | Insertion* |

E-E306K

| Protein | Pre-Launch: Nucleotide Change | Amino Acid Change | Passage 4: Nucleotide Change | Amino Acid Change |
|---|---|---|---|---|
| M | | | T936G (7.9%)* | na |
| M | | | C946G (5.4%)* | M - L66V |
| M | | | C952A (5.3%)* | M - L68I |
| E | G1891A | E306K | G1891A (80.3%) (10.3%G, 9.4%C) | E - E306K |
| E | A1893G | | A1893G (89.5%) (7.4% T) | |
| E | | | A1894G (5.3%) | E - K307E |
| E | | | A2143G (5.8%) | E - K390E |
| E | | | G2145T (10.8%) | E - K390N |
| NS1 | | | A3014C (6.6%) | NS1 - D90A |
| NS1 | | | C3349 +A (8.9%) | Insertion* |
| NS5 | | | C7776A (6.5%) | na |

NS1-AAA

| Protein | Pre-Launch: Nucleotide Change | Amino Acid Change | Passage 4: Nucleotide Change | Amino Acid Change |
|---|---|---|---|---|

| | | | | |
|---|---|---|---|---|
| PrM | | | T543C (99.8%) | na |
| M | | | T936G (8.9%)* | na |
| M | | | C946G (6%)* | M – L66V |
| M | | | C952A (5.1%)* | M – L68I |
| NS1 | A2863A | NS1 - N130A | A2863A (99.4%) | NS1 - N130A |
| NS1 | A2864C | | A2864C (99.6%) | |
| NS1 | A2998G | NS1 - N175A | A2998G (99.7%) | NS1 - N175A |
| NS1 | A2999C | | A2999C (99.8%) | |
| NS1 | A3034G | NS1 - N207A | A3034G (99.7%) | NS1 - N207A |
| NS1 | A3095C | | A3095C (99.6%) | |
| NS1 | | | C3449 +A (6%) | insertion* |

<u>NS1-QQAAA</u>

| | Pre-Launch: | | Passage 4: | |
|---|---|---|---|---|
| Protein | Nucleotide Change | Amino Acid Change | Nucleotide Change | Amino Acid Change |
| M | | | T936G (9%)* | |
| M | | | C946G (5.7%)* | M – L66V |
| M | | | T950C (5.4%)* | M – L67S |
| NS1 | C | NS1 - N130Q | A2863C (99.7%) | NS1 - N130Q |
| NS1 | G | | C2865G (99.8%) | |
| NS1 | G | NS1 - H131Q | C2868G (99.9%) | NS1 - H131Q |
| NS1 | G | NS1 - T132A | A2869G (99.7%) | NS1 - T132A |
| NS1 | G | | T2871G (98.4%) | |
| NS1 | G | NS1 - N175A | A2998G (99.8%) | NS1 - N175A |
| NS1 | C | | A2999C (99.7%) | |
| NS1 | G | NS1 - N207A | A3034G (99.4%) | NS1 – N207A |
| NS1 | C | | A3095C (99.8%) | |
| NS1 | | | A3097C (20%) | NS1 - T208P |
| NS1 | | | G3416A (6.6%) | NS1 – R314Q |
| NS5 | | | G7798T (99.8%) | NS5 – A39S |
| NS5 | | | A8307G (8.1%) | na |
| 3'UTR | | | A10602 – (15.6%) | deletion |

<u>NS2A-A30P</u>

| | Pre-Launch: | | Passage 4: | |
|---|---|---|---|---|

| Protein | Nucleotide Change | Amino Acid Change | Nucleotide Change | Amino Acid Change |
|---|---|---|---|---|
| NS1 | | | C2478 – (19.8%) | deletion |
| NS1 | | | C2480 + C (18.8%) | insertion |
| NS1 | | | G2481 A (18%) | na |
| NS2A | G3619C | NS2A – A30P (100%) | G3619C | NS2A – A30P (100%) |

NS4B-P41G

| | Pre-Launch: | | Passage 4: | |
|---|---|---|---|---|
| Protein | Nucleotide Change | Amino Acid Change | Nucleotide Change | Amino Acid Change |
| PrM | | | A838G (8.1%) | PrM – M122V |
| E | | | C1342T (48.8%) | na |
| NS1 | | | C2839T (80.6%) | na |
| NS1 | | | A3402C (49.3%) | na |
| NS1 | | | C3449 + A (11.1%) | Insertion |
| NS2A | | | C3621T (9.5%) | na |
| NS3 | | | C5829C (25.7%) | na |
| NS4B | G | NS4b - P41G | C7030G (100%) | NS4B – P41G |
| NS4B | G | | C7031G (80.2%) | |
| | | | C (19.7%) | NS4B – P41A |
| NS4B | | | A7183C (73.2%) | NS4B - T92P |
| NS4B | | | T7213C (25.7%) | NS4B – F102L |
| NS4B | | | C7265T (25.5%) | NS4B – A119V |
| NS4B | | | A7307G (12.3%) | NS4B – Q133R |
| NS4B | | | C7589T (13.3%) | NS4B – T227I |
| NS4B | | | C7640T (6.7%) | NS4B – A244V |
| NS5 | | | T8274C (48.6%) | na |
| NS5 | | | C8607T (23.6%) | na |
| 3'UTR | | | G10415 + T (15.1%) | Insertion |

NS4B-C105S

| | Pre-Launch: | | Passage 4: | |
|---|---|---|---|---|
| Protein | Nucleotide Change | Amino Acid Change | Nucleotide Change | Amino Acid Change |
| M | | | T936G (8.3%)* | |
| M | | | C946G (5.6%)* | M – L66V |

| | | | | |
|---|---|---|---|---|
| M | | | T950C (9.1%)* | M – L67S |
| NS1 | | | C3449 +A (9.3%) | insertion* |
| NS4B | G7223C | NS4b – C105S | G7223C (99.7%) | NS4B – C105S |

NS4B-D252G

| Protein | Pre-Launch: Nucleotide Change | Amino Acid Change | Passage 4: Nucleotide Change | Amino Acid Change |
|---|---|---|---|---|
| M | | | T936G (7.7%)* | |
| M | | | C946G (5.5%)* | M – L66V |
| E | | | T1606C (6.1%) | E – F211L |
| NS1 | | | C3449 + (9.9%) | insertion* |
| NS4B | A7664G | NS4BB - D252G | A7664G (25%) | NS4B - D252G |

Table S4. Reference sequences used in sequence alignments for supplementary figure1.

| Virus | Genbank Entry |
|---|---|
| rUSUV-WT Recombinant Clone<br>- Strain TM Netherlands 2016 | PQ041659.1 |
| Usutu virus (USUV), complete cds<br>- NCBI Reference (Vienna 2001) | NC_006551.1 |
| Murray Valley encephalitis virus (MVEV), complete genome.-<br>NCBI Reference Sequence | NC_000943 |
| Japanese encephalitis virus (JEV), genome<br>- NCBI Reference Sequence | NC_001437 |
| West Nile virus lineage 1, complete genome<br>- NCBI Reference Sequence | NC_009942 |
| West Nile virus lineage 2, complete genome<br>-NCBI Reference Sequence | NC_001563 |
| Kunjin virus (KUNV) gene for polyprotein<br>-strain MRM61C | D00246.1 |
| Saint Louis encephalitis virus (SLEV), complete genome<br>-NCBI Reference Sequence | NC_007580 |
| Zika Virus (ZIKV), complete genome<br>-NCBI Reference Sequence | NC_012532.1 |

| | |
|---|---|
| Dengue virus 2 (DENV), complete genome<br>-NCBI Reference Sequence | NC_001474 |
| Yellow fever virus (YFV), complete genome<br>-NCBI Reference Sequence | NC_002031 |
